# Ligand unbinding mechanisms and kinetics for T4 lysozyme mutants from τRAMD simulations

**DOI:** 10.1016/j.crstbi.2021.04.001

**Published:** 2021-05-04

**Authors:** Ariane Nunes-Alves, Daria B. Kokh, Rebecca C. Wade

**Affiliations:** a Molecular and Cellular Modeling Group, Heidelberg Institute for Theoretical Studies, Schloss-Wolfsbrunnenweg 35, 69118, Heidelberg, Germany; b Center for Molecular Biology (ZMBH), DKFZ-ZMBH Alliance, Heidelberg University, Im Neuenheimer Feld 282, 69120, Heidelberg, Germany; c Interdisciplinary Center for Scientific Computing (IWR), Heidelberg University, Im Neuenheimer Feld 205, Heidelberg, Germany

**Keywords:** Ligand dissociation pathways, Drug design, Ligand-protein binding kinetics, Molecular dynamics simulations, Protein engineering, Residence time

## Abstract

The protein-ligand residence time, τ, influences molecular function in biological networks and has been recognized as an important determinant of drug efficacy. To predict τ, computational methods must overcome the problem that τ often exceeds the timescales accessible to conventional molecular dynamics (MD) simulation. Here, we apply the τ-Random Acceleration Molecular Dynamics (τRAMD) method to a set of kinetically characterized complexes of T4 lysozyme mutants with small, engineered binding cavities. τRAMD yields relative ligand dissociation rates in good accordance with experiments across this diverse set of complexes that differ with regard to measurement temperature, ligand identity, protein mutation and binding cavity. τRAMD thereby allows a comprehensive characterization of the ligand egress routes and determinants of τ. Although ligand dissociation by multiple egress routes is observed, we find that egress via the predominant route determines the value of τ. We also find that the presence of a greater number of metastable states along egress pathways leads to slower protein-ligand dissociation. These physical insights could be exploited in the rational optimization of the kinetic properties of drug candidates.

## Highlights

• Relative residence times are computed for T4 lysozyme mutant-ligand complexes.
• τ-Random Acceleration Molecular Dynamics provide efficient sampling of unbinding.
• Computed dissociation rates show good agreement with all available measured values.
• Ligand egress via the predominant route determines the value of the residence time.
• The presence of metastable states along egress pathways slows down dissociation.

## Graphical abstract

**Figure:**
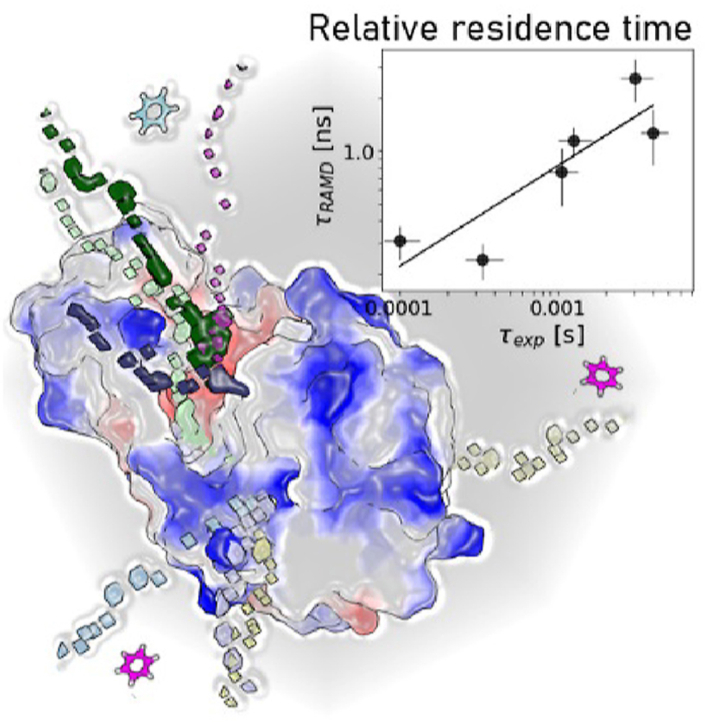

## 1 Introduction

The residence time of a ligand-protein complex (τ, given by the inverse of the dissociation rate: 1/k_off_) has become an important parameter in drug design, since for some targets, it shows a stronger correlation than the binding affinity with *in vivo* drug efficacy (Bernetti et al., 2019; Copeland, 2016; Romanowska et al., 2015; Schuetz et al., 2017). However, the determinants of protein-ligand residence times are not well understood. Moreover, the prediction of τ by molecular dynamics (MD) simulation is challenging, in particular because of the timescales involved. Although simulations of protein systems may now routinely extend to microseconds, they are short compared to typical values of τ for drug-like molecules. To overcome this problem, many computational methods to enhance sampling of ligand unbinding during MD simulations have been proposed (Bruce et al., 2018; Nunes-Alves et al., 2020). However, it remains to be determined to what extent such approaches, which in some cases employ non-equilibrium perturbations, can correctly capture mechanistic details of ligand egress routes.

**Fig. 1:**
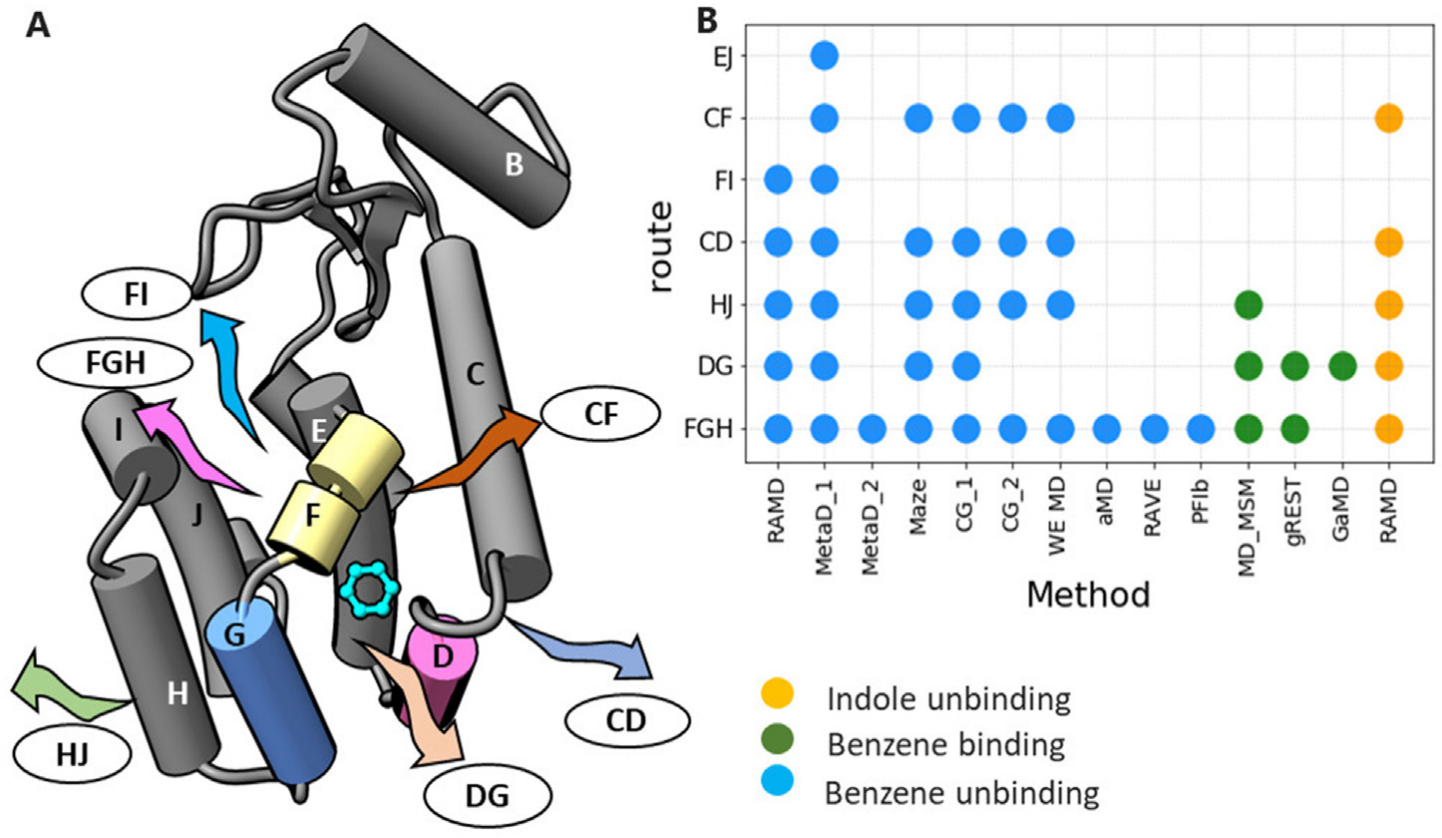
*Egress routes observed for benzene and indole dissociation from the T4L:L99A mutant. (A) Cartoon representation of the protein with helices labelled. Egress routes are denoted by the helices lining them. (B) Ligand binding and unbinding routes observed in recent computational studies are indicated by circles colored by simulation type. Methods: MetaD_1* (Capelli et al., 2019) *and MetaD_2* (Wang et al., 2016) *- metadynamics, Maze* (Rydzewski and Valsson, 2019)*, CG_1* (Dandekar and Mondal, 2020) *and CG_2* (Souza et al., 2020) *- coarse-grained, WE MD* (Nunes-Alves et al., 2018) *– weighted ensemble MD, aMD* (Feher et al., 2019) *- accelerated MD, RAVE* (Lamim Ribeiro and Tiwary, 2019) *- Reweighted autoencoded variational Bayes for enhanced sampling; PFIb* (Wang et al., 2019) *- Past-future information bottleneck, MD_MSM* (Mondal et al., 2018) *– conventional MD and Markov State Model, gREST* (Niitsu et al., 2019) *- generalized replica exchange with solute tempering, GaMD* (Miao et al., 2015) *– Gaussian accelerated MD.*

T4 lysozyme (T4L) mutants that contain engineered small artificial cavities that can accommodate benzene and indole derivatives have long served as model systems for investigating the fundamental mechanisms underlying protein-small molecule interactions and for benchmarking computational methods (Eriksson et al., 1992a, 1992b). Remarkably, no less than thirteen computational studies have been published since 2018 by different research groups in which methods based on MD simulation were used to identify paths from a buried cavity to the T4L exterior and to try to characterize the ligand binding and unbinding processes energetically and kinetically (see Fig. 1, review Nunes-Alves et al., 2020 and recent papers of Capelli et al., 2019; Dandekar and Mondal, 2020; Feher et al., 2019; Lamim Ribeiro and Tiwary, 2019; Lotz and Dickson, 2020; Mondal et al., 2018; Niitsu et al., 2019; Nunes-Alves et al., 2018; Rydzewski, 2020; Rydzewski and Valsson, 2019; Souza et al., 2020; Wang et al, 2018, 2019).

Here, we apply the τRAMD (Kokh et al, 2018, 2019) procedure to compute relative τ values for a set of T4L-ligand complexes with the goals of (1) assessing the ability of the procedure to compute accurate relative τ values, and (2) comprehensively characterizing the ligand egress routes and identifying the determinants of residence times. In the τRAMD procedure, relative τ values are computed from the ligand dissociation times observed in a set of random acceleration MD (RAMD) trajectories. In RAMD, a randomly oriented force is applied adaptively to the ligand during an MD simulation to enhance the rate of ligand unbinding. We find that, due to its computational efficiency and accuracy, τRAMD, for the first time, enabled a complete characterization of all T4L mutant complexes with experimental kinetic data available. It yielded a good correlation between computed and experimental residence times for the set of measured binding kinetic data for T4L mutant complexes, which includes data measured under different environmental conditions for different ligands, and for different protein mutants with differing binding cavities. Furthermore, the mechanistic insights obtained from the τRAMD simulations allow us to understand how the presence of metastable states along ligand egress paths can affect residence times and how good estimates of protein-ligand residence times can be obtained without having to sample all the egress paths.

## 2 Results and discussion

### 2.1 τRAMD accurately predicts relative residence times for benzene and indole bound to T4L mutants

τRAMD was used to generate dissociation trajectories and to compute relative residence times (as described in Computational Methods in the Supporting Information) for indole and benzene bound to the buried cavity in the L99A mutant of T4L (T4L:L99A), and benzene bound to two additional T4L mutants: T4L:M102A and T4L:F104A (at 20 ​°C). Additionally, the dissociation of benzene from T4L:L99A was simulated at 10 and 30 ​°C to compare with the experimental measurements in Feher et al. (1996).

**Fig. 2:**
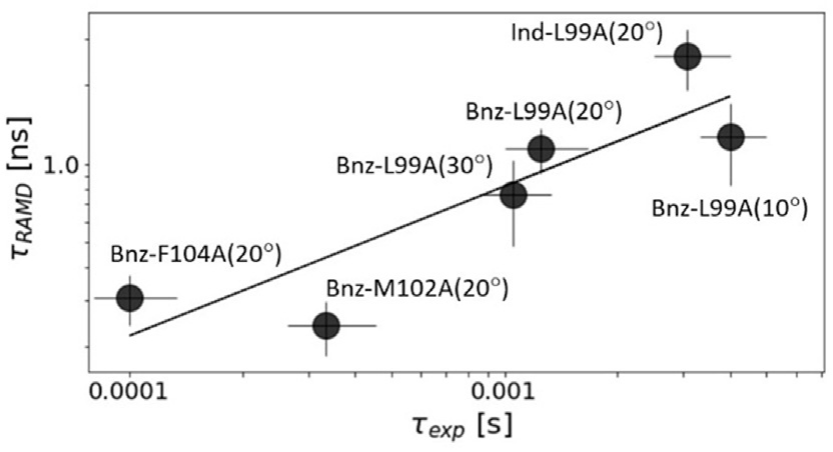
Comparison of computed (τ_RAMD_) and measured (τ_exp_) residence times for benzene and indole for three T4L mutants at 10, 20 and 30 ​°C. Values (which are also given in Table S1) are plotted on the logarithmic scale and a linear fitting of computed to experimental data with R^2^ ​= ​0.78 is shown by the line. For benzene bound to T4L:F104A, τ_exp_ ​< ​10^−4^ ​s and the error bar is defined as 25% (estimated from the uncertainty of the other measurements).

The computed relative residence times show a remarkably good correlation with experimental data (R^2^ ​= ​0.78), with a mean unsigned error of about 38% of the experimental τ values, see Fig. 2. Notably, τRAMD captures the trends in residence time despite the different determinants of τ, which does not correlate with the equilibrium dissociation constant for these systems (Table S1).

### 2.2 The populations of the unbinding paths depend on the binding site, ligand type and temperature

To explore the ligand egress pathways, we generated protein-ligand interaction fingerprints (IFPs) for the last 300 snapshots (i.e. 0.3 ns) of each RAMD trajectory, which generally encompass the last part of the ligand motion in its bound state and the complete ligand unbinding process (see Computational Methods in Supporting Information for details). We first extracted final frames with non-zero protein-ligand IFPs from the dissociation trajectories and carried out hierarchical clustering of these frames for each complex type.

**Fig. 3:**
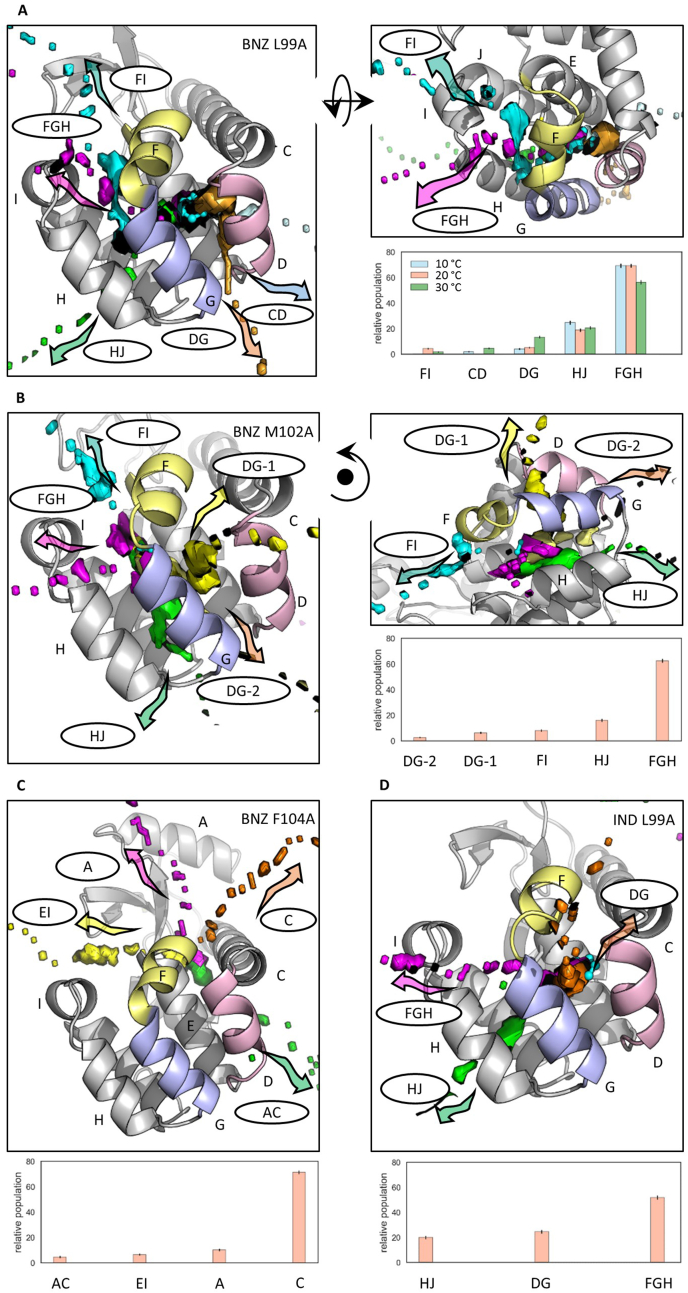
Paths and their relative populations observed in RAMD trajectories for benzene dissociation from T4L:L99A at 10, 20 and 30 ​°C (A), benzene dissociation from T4L:M102A (B) and T4L:F104A (C), and indole dissociation from T4L:L99A (D) at 20 ​°C. Averages and standard deviations of the relative populations were calculated using bootstrapping. The main dissociation paths observed were obtained from hierarchical clustering and are labelled according to the helices that they pass between. Each path is represented by one or two arbitrarily chosen dissociation trajectories (that belong to the corresponding cluster) displayed as isosurfaces of the population density obtained by mapping positions of the ligand center of mass in all frames of the trajectory onto a 3D grid. The directions of dissociation are indicated by arrows. The protein is shown in cartoon representation with helices labelled by letters and helices D, F and G colored pink, yellow and blue, respectively. The ligands are shown in cyan ball-and-stick representation in their bound position.

For benzene dissociating from T4L:L99A, five egress routes were identified (shown in Fig. 3A and Fig. S1). These were also reported in most of the previous simulation studies (see Fig. 1), with the FGH route (routes are named by the helices lining them) being clearly predominant at all conditions. The FGH route was the only one recorded in several publications using metadynamics, machine learning and aMD approaches (Feher et al., 2019; Lamim Ribeiro and Tiwary, 2019; Wang et al, 2016, 2019). However, the FGH route can be subdivided further by lowering the threshold for hierarchical clustering (see Fig. S1 and Ref (Capelli et al., 2019).). Two additional pathways, CF and EJ, were observed with low populations in several enhanced sampling simulations (Capelli et al., 2019; Dandekar and Mondal, 2020; Nunes-Alves et al., 2018; Rydzewski and Valsson, 2019) but were not observed here for benzene although the CF pathway was observed for indole (see Fig. S5).

The bound position of benzene in T4L:M102A is slightly shifted towards A102 (away from helix D) relative to its position in T4L:L99A. Accordingly, the egress routes are very similar to those for T4L:L99A: FGH, DG, HJ and FI, except for CD, which is not observed. The DG route can be further divided into DG-1 and DG-2, depending on whether the dissociation direction is perpendicular or parallel to the D and G helices (Fig. 3B and S2).

In T4L:F104A, benzene occupies another binding site that is large and highly solvent-exposed. The four main paths found lead directly from the large open cavity that is lined by helices A, C and E. Only a few trajectories were observed in the opposite direction, via pathway AC (Fig. 3C and S3).

The dissociation routes of indole from the same bound position in T4L:L99A as benzene are quite similar (Fig. 3D and S4). However, path CD, which was observed for benzene, had a low population for indole, likely due to its larger size (Fig. S5). Path FI was not identified for indole, but could be considered as part of the wider path FGH (Fig. S4).

### 2.3 Different egress routes have similar ligand dissociation times

Remarkably, despite the large differences in population, there are only small differences in the average dissociation times computed for all the pathways (see Figs. S1–4). These results agree with other studies, in which similar preliminary kinetic rates (Nunes-Alves et al., 2018) or unbinding times (Rydzewski and Valsson, 2019) were computed for the different paths. This suggests that a reasonably accurate prediction of the unbinding rate for benzene can be made without exhaustive pathway sampling, as long as the main path, FGH, is sampled, as done, for example, in Mondal et al. (2018).

### 2.4 Visiting multiple intermediate metastable states makes dissociation slower

**Fig. 4:**
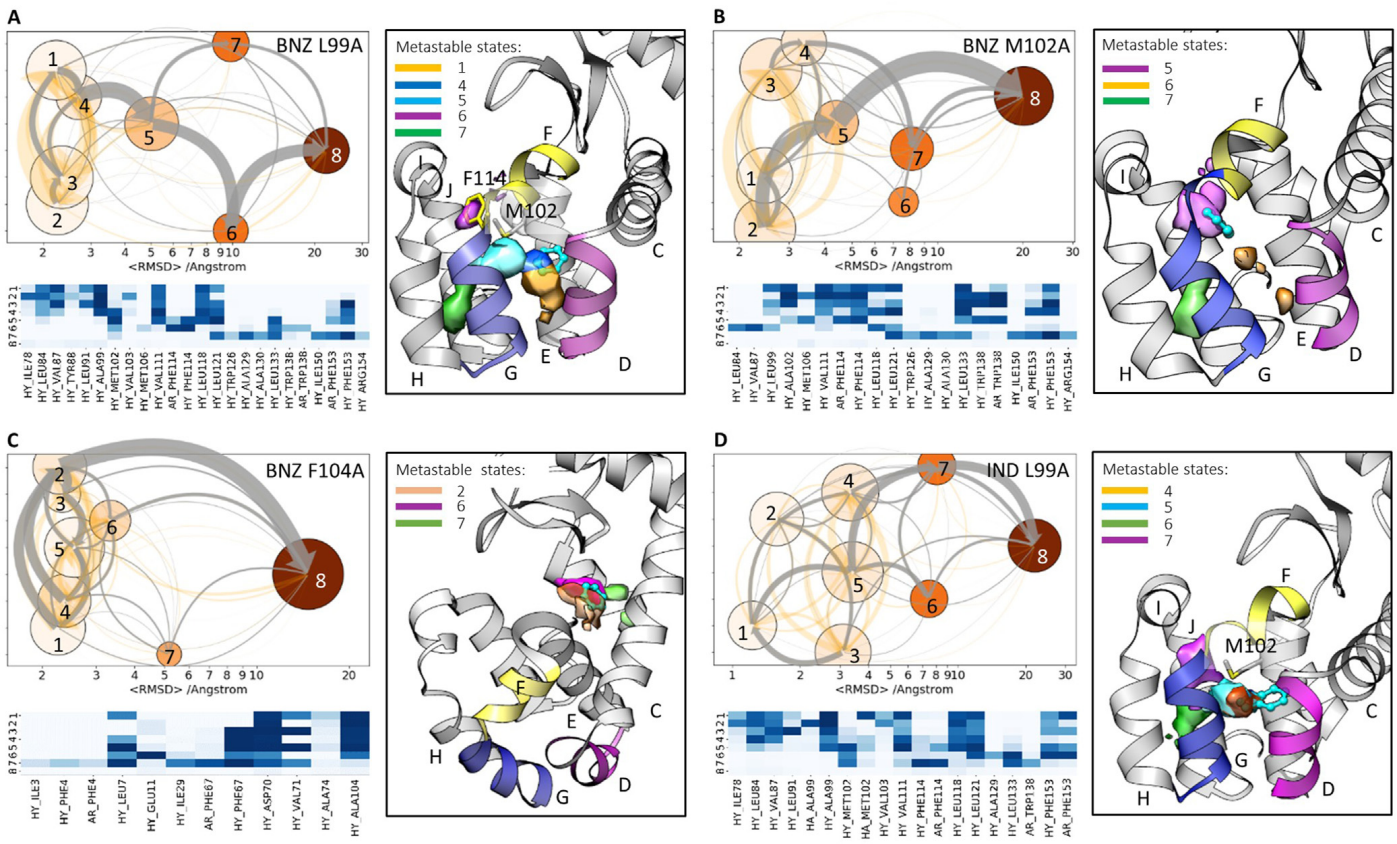
Analysis of benzene unbinding from T4L:L99A (A), T4L:M102A (B) and T4L:F104A (C), and indole unbinding from T4L:L99A (D) in RAMD trajectories. Clusters were defined by clustering of frames from egress trajectories in IFP space. Dissociation pathways are shown in a graph-representation; each node represents a cluster or metastable state that is colored and placed according to increasing mean RMSD of the ligand in the cluster from in the starting complex; the node size denotes the cluster population; transitions between nodes are indicated by arrows for simulations at 20 ​°C (transitions for simulations at 10 and 30 ​°C are shown in Fig. S6): the net transition flux between nodes is shown by gray arrows with their thickness proportional to the flux magnitude; the transitions between states are shown by orange arrows with their thickness proportional to the number of transition events. Some clusters are displayed as isosurfaces of the ligand center of mass population density mapped onto the 3D grid. Helices D, F and G are shown in pink, yellow and blue, and the ligand is shown in cyan ball-and-stick representation. The heat maps show the composition of the clusters, in terms of ligand-protein contacts (color pallet from white to dark-blue indicates increasing contribution). HY: hydrophobic interactions. AR: aromatic interactions.

Metastable states on the dissociation pathways for each complex were identified using k-means clustering of the IFPs computed for the last 300 frames of each dissociation trajectory (see Supporting Information for details). The pattern of the metastable states for the benzene-T4L:L99A system is very similar for all temperatures simulated (Fig. 4A and S6): there are two metastable states with relatively low populations at average RMSD values of about 10 ​Å (clusters 6 and 7; cluster 8 corresponds to the unbound state), which are intermediates on the dissociation paths FGH and HJ (Fig. 3A), and there is a highly populated (often visited) metastable state 5 ​at an RMSD of ~5 ​Å where benzene is located close to the side-chain of M102. All other states (average RMSD ​< ​3 ​Å, clusters 1–4 in Fig. 4A) can be assigned to variations of the bound state. The dissociation flow can be described as ligand transitions from metastable state 4 to 5, 6, and then complete dissociation (gray arrows in Fig. 4A). Direct transitions from the bound states 1 and 3 to the dissociated state (via paths CD and DG, respectively) and from 5 to 7 to dissociation (path HJ) are also observed, albeit with a lower probability.

The present results are consistent with previous conventional MD simulations of ligand binding accompanied by a Markov State Model analysis, where two main intermediate macrostates were identified (Mondal et al., 2018): MS1 between helices G and H, and MS2 covering the region between helices D and G, which is represented by clusters 1–4 close to the bound state in our simulations (see Fig. 4A). MS1, an intermediate on the main association pathway, is more spread out and includes regions occupied by clusters 5, 6 and 7. MS1 is the intermediate state with the most flux during ligand binding, while the alternative binding path through MS2 (analogous to dissociation route DG from cluster 1) was observed much less, in agreement with our analysis. Remarkably, although cluster 6 is an intermediate on the main dissociation flow (see path FGH in Fig. 3A and the dissociation network in Fig. 4A), it is slightly less populated and more spatially localized than cluster 7. Gating by F114 on the dissociation pathway is likely to be the main reason for the ligand spending time in cluster 6. However, flipping of F114 is rather fast and thus does not slow down ligand dissociation significantly, whereas squeezing between helices H and J (i.e. via metastable state 7) is notably slower, making dissociation path HJ less populated.

Enhanced sampling simulations of benzene binding by GaMD (Miao et al., 2015) and gREST (Niitsu et al., 2019) revealed only the metastable states between helices D and G (i.e. clusters 1–4). In unbinding simulations using aMD (Feher et al., 2019), intermediate states between helices G and H (cluster 5) and between helices F and H (resembling cluster 6 but shifted closer to helix H) were identified. Thus, not all of the metastable states identified in conventional MD and RAMD simulations were revealed by these enhanced sampling methods. Moreover, small changes in protein structure were associated with ligand unbinding (see Fig. S7).

The dissociation flow for indole has a similar pattern to benzene in RAMD trajectories, albeit with a larger variety of bound states (clusters 1–5, Fig. 4D). Indeed, the indole residence time in T4L:L99A at 20 ​°C is comparable to that for benzene at 10 ​°C. The temperature difference is consistent with indole being slightly larger and needing to squeeze through the narrow channel gated by F114.

In contrast to T4L:L99A, for the M102A and F104A mutants, the pattern of benzene dissociation trajectories is different: the main flow leads either directly from the bound state to dissociation (T4L:F104A, Fig. 4C) or through the intermediate state located in the vicinity of the bound one (T4L:M102A, Fig. 4B; cluster 5 is close to F114). Accordingly, the dissociation time of benzene from both mutants is notably shorter than from T4L:L99A.

Thus, the benzene residence times are related to the number of intermediate metastable states for egress from the three mutants in the RAMD simulations (Fig. 4A–C). Each metastable state can be associated with a subsequent transition barrier along the dissociation path, and therefore, a corresponding prolongation of the dissociation time.

## 3 Conclusions

We have presented a computational characterization of the unbinding processes for a set of complexes of T4L mutants with benzene and indole. We find that τRAMD provides very good agreement between computed relative unbinding rates and experimental data obtained for distinct conditions: different ligands, different mutants with different binding cavities, and different temperatures. We find that for benzene dissociation from T4L:L99A, there is one dominant egress path, FGH, which explains why only this pathway was found in many computational studies and indicates that accurate dissociation rates can be computed for this system even if just the main egress route is sampled. Our study also showed that longer τ is associated with more complex dissociation pathways with multiple intermediate metastable states (as seen for indole and benzene dissociating from T4L:L99A), in contrast to the one-step dissociation observed for complexes with shorter τ (benzene – T4L:M102A and T4L:F104A). The physical insights revealed here can be used in the rational optimization of the kinetic properties of drug candidates.

## CRediT authorship contribution statement

**Ariane Nunes-Alves:** Conceptualization, Methodology, Formal analysis, Investigation, Writing – original draft, Writing – review & editing, Visualization. **Daria B. Kokh:** Conceptualization, Methodology, Software, Formal analysis, Investigation, Writing – original draft, Writing – review & editing, Visualization. **Rebecca C. Wade:** Conceptualization, Formal analysis, Writing – review & editing, Visualization, Supervision.

## Declaration of competing interest

The authors declare that they have no known competing financial interests or personal relationships that could have appeared to influence the work reported in this paper.
