# Supplementary material for: Ligand unbinding mechanisms and kinetics for T4 lysozyme mutants from τRAMD simulations
Source: Curr Res Struct Biol. 2021 May 4;3:106–11. doi: 10.1016/j.crstbi.2021.04.001 (PMC8244441; doi:10.1016/j.crstbi.2021.04.001)
Supplement: Multimedia component 2 [file mmc2.docx]

**Supporting Information**

Ligand Unbinding Mechanisms and Kinetics for T4 Lysozyme Mutants from τRAMD Simulations

Ariane Nunes-Alves^a,b^, Daria B. Kokh^a^, Rebecca C. Wade^a,b,c*^

^a^Molecular and Cellular Modeling Group, Heidelberg Institute for Theoretical Studies, Schloss-Wolfsbrunnenweg 35, 69118 Heidelberg, Germany

^b^Center for Molecular Biology (ZMBH), DKFZ-ZMBH Alliance, Heidelberg University, Im Neuenheimer Feld 282, 69120 Heidelberg, Germany

^c^Interdisciplinary Center for Scientific Computing (IWR), Heidelberg University, Im Neuenheimer Feld 205, Heidelberg, Germany.

Corresponding Author

*Rebecca.Wade@h-its.org

**Computational Methods**

**A. Molecular dynamics simulations**

The structures of the complexes of benzene bound to the T4L:L99A, T4L:M102A and T4L:F104A mutants were obtained from the PDB files 3HH4 [1], 220L [2] and 227L [2], respectively. The crystal structure of indole bound to T4L:L99A was obtained from PDB file 185L [3]. Protonation states were assigned using pdb2pqr [4,5] at pH 5.5 to mimic kinetic experiments [6]. Crystallographic water molecules were maintained and crystallization molecules were removed.

The τRAMD method [7] was used to compute relative residence times. The protocol for the setup of each system and for molecular dynamics simulation was described in detail previously [7] and is therefore described briefly here. The AMBER ff14SB force field [8] was used for the protein and the GAFF force field [9] for the ligand. RESP [10,11] partial atomic charges for ligands were obtained using molecular electrostatic potentials from quantum mechanical calculations performed at the HF level with HF/6-31G** basis set using GAMESS [12]. Each system was solvated in a periodic box of TIP3P water molecules with a distance of 10 Å from the solute to the box edge using tleap. 7 Na^+^ ions and 16 Cl^-^ ions were added to achieve neutrality and an approximate ionic strength of 50 mM, which mimics the conditions of the kinetic experiments. The system was energy minimized and gradually heated to 293 K in 1 ns with the Langevin thermostat and harmonic restraints of 50 kcal/(mol Å^2^) on all non-hydrogen atoms of the protein and the ligand using the AMBER14 software [13]. Pressure was adjusted to 1 atm in a further 2 ns simulation using the Berendsen barostat. Then the system was run for 2 more nanoseconds without restraints and the final snapshot was used as input for heating and equilibration simulations carried out with the NAMD software [14]. Heating from 0 to 293 K was performed for 6 ns in the NVT ensemble using the Langevin thermostat. Then equilibration was performed for 20 ns in the NPT ensemble using the Langevin thermostat and the Nosé–Hoover barostat for temperature (293 K) and pressure (1 atm) control, respectively. Eight replicas of equilibration simulations were performed for each system. The last snapshot of each replica was used to simulate ligand dissociation. For this purpose, the RAMD method [15] was applied, in which an additional force with a magnitude of 4 kcal/(mol Å) and a random direction was applied to the center of mass (COM) of the ligand in MD simulations. Every 100 fs, the force direction was changed randomly if the ligand COM did not move further than 0.025 Å and was retained otherwise. For each of the eight equilibration replicas, 15 RAMD dissociation trajectories were generated, resulting in a total of 120 trajectories for each system. Simulations were stopped and the ligand considered to be dissociated when the distance between the COMs of the ligand and the protein was greater than 40 Å. The time required for ligand dissociation in each trajectory was stored and the recorded dissociation times for the set of trajectories were used to compute the relative residence times.

**B. Analysis protocol**

The protein-ligand interaction fingerprints, IFPs, were computed for the last 300 frames saved at intervals of 1 ps of each RAMD trajectory using the MD-IFP method described in ref. [16]. The results were similar when the last 500 snapshots were used (**Figure S8**). The IFPs analysed for benzene and indole included aromatic interactions, hydrogen bonds, and hydrophobic interactions. Additionally, the coordinates of the ligand COM and the ligand RMSD from the starting equilibrated complex were stored for each frame. The computed IFPs for the last 300 frames of each trajectory were combined in one IFP binary table for each complex (the simulations of dissociation of the benzene-T4L:L99A complex at different temperatures were analysed together), with an entry of 1 if a contact was observed and zero if it was not observed. All frames were then split into 8 clusters based on their IFP composition by using a k-means algorithm, which provided the most populated states of the ligand in the IFP space (The simulations of the benzene-T4L:L99A complex at all three temperatures were analyzed together). Each cluster represents either the bound, a metastable, or the unbound state, which can be distinguished by the average RMSD of the ligand non-hydrogen atoms from their position in the starting complex. The position of the ligand COM mapped onto a 3D grid (with a spacing of 1 Å) was used to generate and display the COM density distribution for each cluster.

Additionally, the last frames of each RAMD dissociation trajectory that had IFP vectors containing at least 2 protein-ligand contacts were combined into a dissociation IFP set that was then clustered using a hierarchical clustering procedure. For each system, a variable clustering threshold was chosen in order to obtain 3-5 clusters corresponding to the main unbinding paths. The dissociation paths were then displayed by plotting the ligand COM density distribution for all trajectories or one representative trajectory in the corresponding cluster. Averages and standard deviations of the relative path populations and of residence times were calculated using bootstrapping. Bootstrapping was performed resampling a population with 90% of the original size 500 times.

**Table S1. *Experimental values of*** *K_D_, k_off_* ***and*** *k_on_* ***for the T4L-ligand complexes studied from Ref.*** [6]***.*** *Comparison of these values* *shows that the mechanisms determining the length of the residence times differ among the systems studied.^a^*

| **T4L mutant** | **Ligand** | **Temperature (°C)** | K_D_ (mM) | k_off_ (s^-1^) | k_on_ (10^6^ M^-1^s^-1^)^b^ | τ_RAMD_ [ns] |
| --- | --- | --- | --- | --- | --- | --- |
| L99A | Benzene | 10 | 0.3±0.2 | 250±50 | 0.83 | 1.27±0.44 |
|  |  | 20 | 0.8±0.12 | 800±200 | 1.00 | 1.15±0.22 |
|  |  | 30 | 1.1±0.11 | 950±200 | 0.86 | 0.75±0.27 |
| M102A |  | 20 | 0.8±0.1 | 3000±800 | 3.75 | 0.24±0.05 |
| F104A |  | 20 | 1.3±0.05 | >10^4^ | >7.7 | 0.31±0.06 |
| L99A | indole | 20 | 0.35±0.06 | 325±75 | 0.92 | 2.60±0.68 |

*^a^The difference in τ between benzene and indole for binding to T4L:L99A at 20 °C can be ascribed solely to a relative stabilization of the bound state in the indole complex as both K_D_ and k_off_ differ by about the same factor (2.3). In contrast, the difference in τ for benzene binding to T4L:L99A and to T4L:M102A can be ascribed solely to lowering of the transition barrier for unbinding since the K_D_ is the same for both mutants while the k_off_ differs by a factor of 3.75. On the other hand, the difference in τ for benzene binding to the more exposed cavity of T4L:F104A versus the other mutants appears to be due to both a destabilization of the bound state and a lowering of the transition barrier.*

*^b^ Computed as k_on_ = k_off_ /K_D_*

**Supplementary Figures**

**
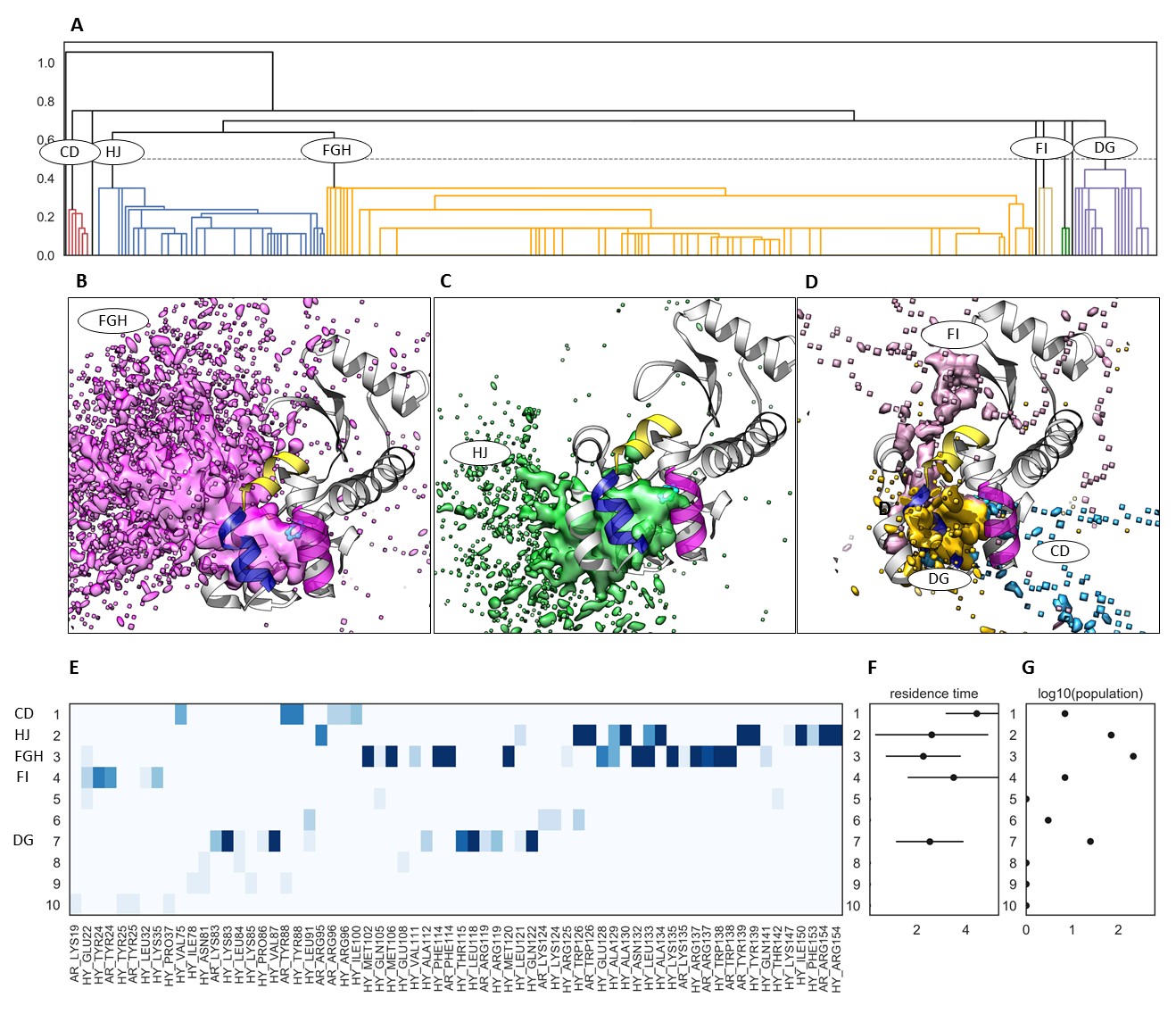
**

***Figure S1.*** *Analysis of egress routes for benzene dissociation from T4L:L99A in RAMD simulations at three temperatures: 10, 20 and 30 °C. (A) Results of hierarchical clustering. (B-D) Display of the most populated clusters (populated by more than one snapshot (each representing one trajectory)). (E) Cluster composition in terms of IFPs. (F) Computed residence times (in nanoseconds) for the highly-populated clusters shown in (E). (G) The corresponding cluster populations shown on a logarithmic scale.*

**
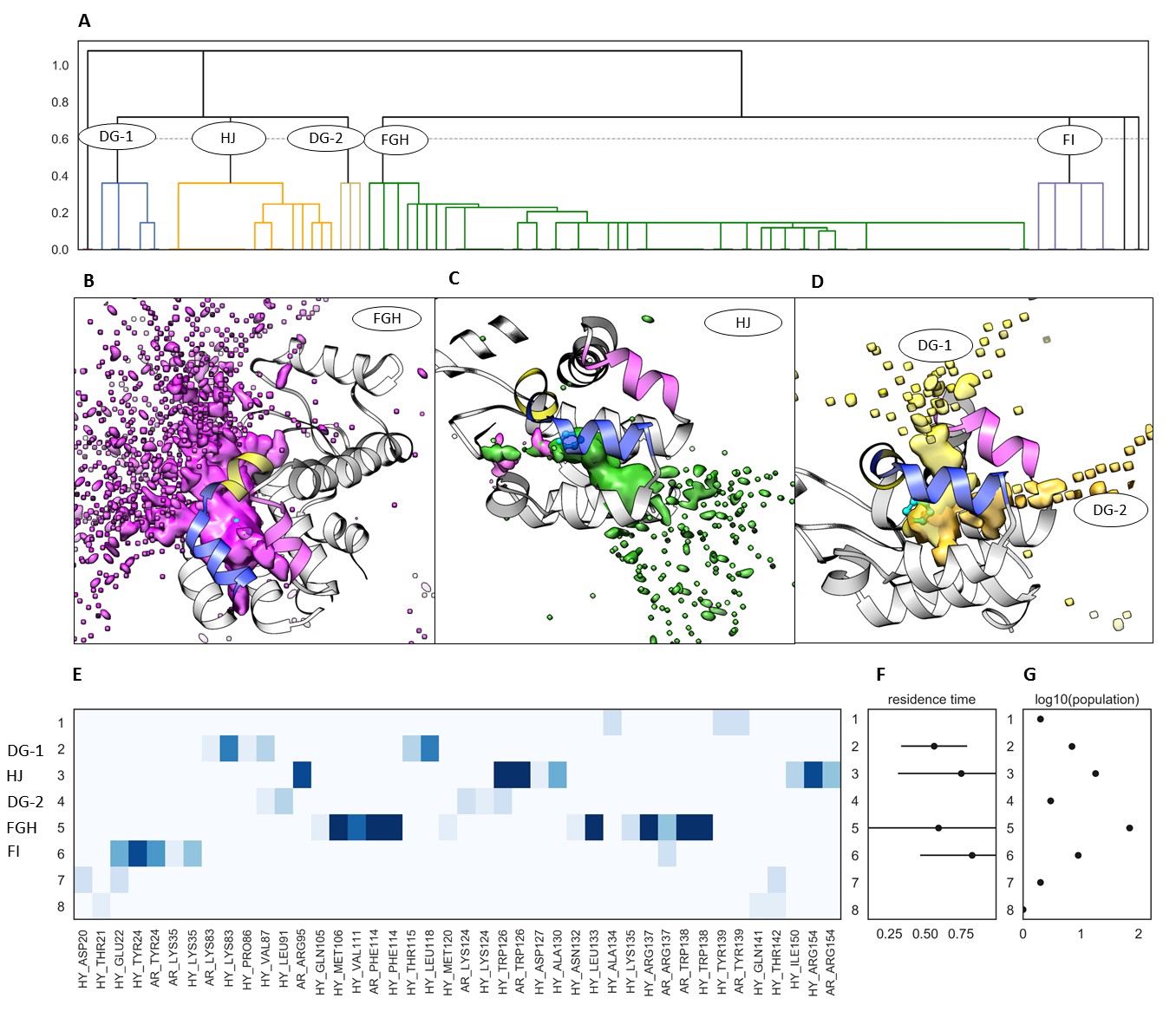
**

***Figure S2.*** *Analysis of egress routes for benzene dissociation from T4L:M102A. Legend as for Figure S1*.

**
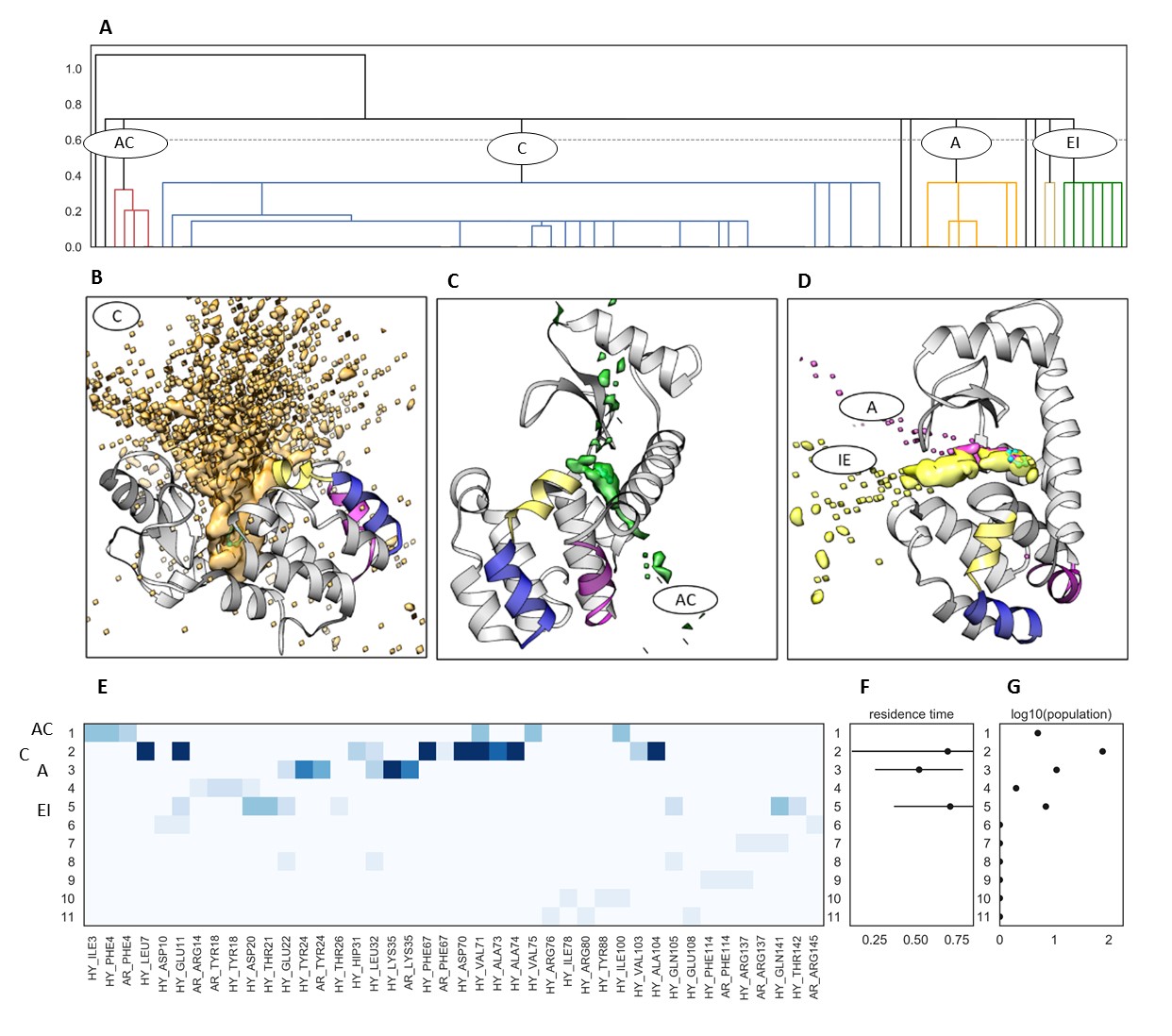
**

***Figure S3.*** *Analysis of egress routes for benzene dissociation from T4L:F104A. Legend as for Figure S1.*

**
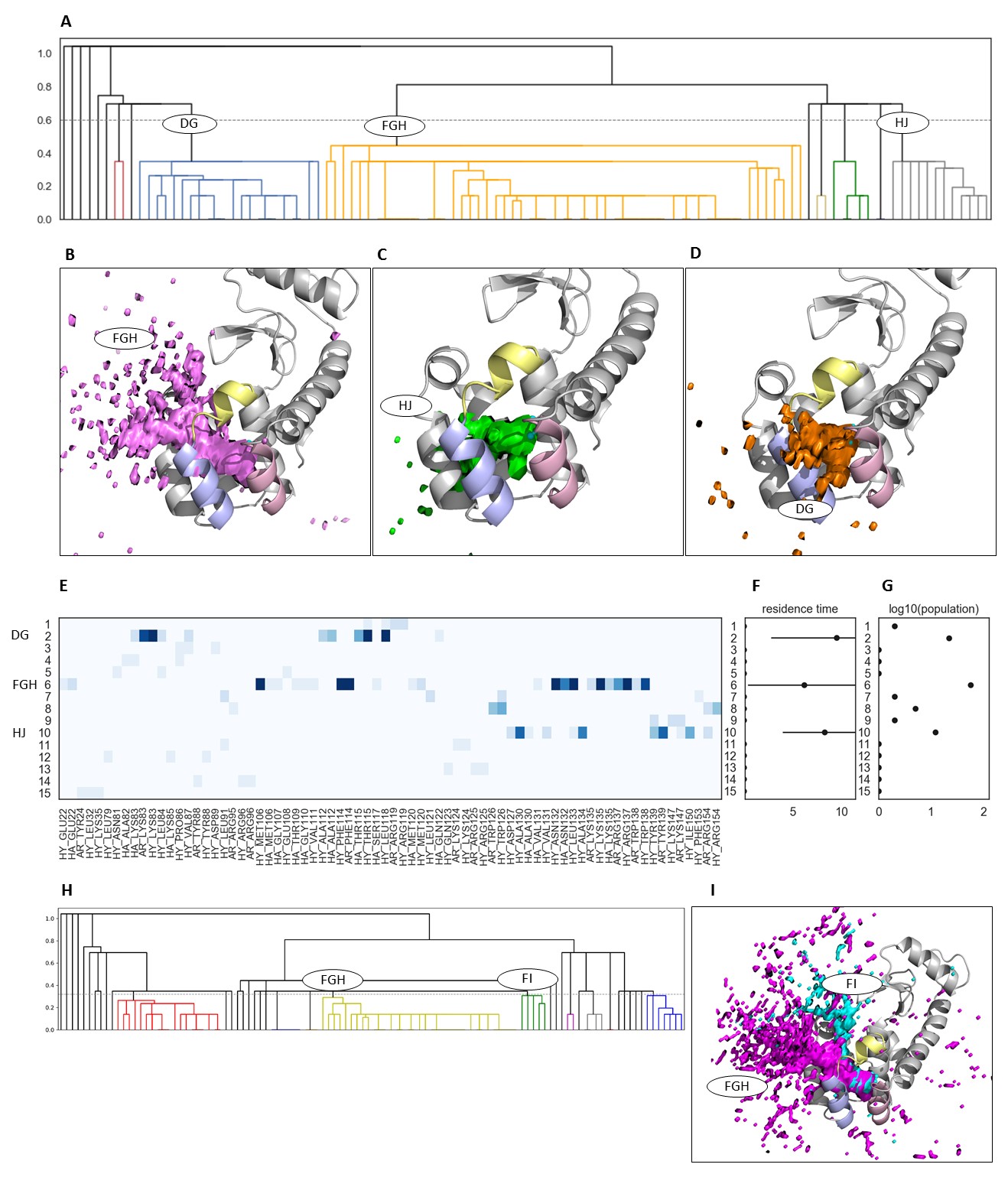
**

***Figure S4.*** *Analysis of egress routes for indole dissociation from T4L:L99A. (A-G) Legend as for Figure S1. (H-I) Results for a smaller cutoff in the hierarchical clustering. (H) Results of hierarchical clustering. (I) Display of clusters for paths FGH and FI.*

**
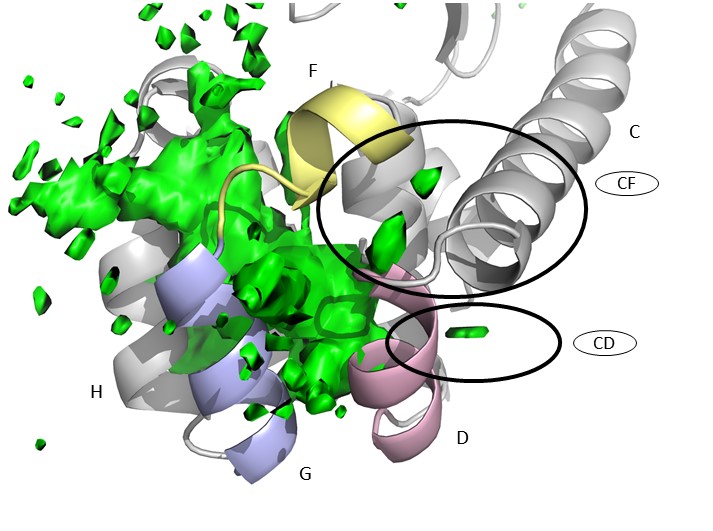
**

***Figure S5.*** *All dissociation paths (green) for indole from T4L:L99A are displayed. Paths CD and CF have a low population (< 5%) and are therefore not shown in figure 3 of the main text. The paths are represented by an isosurface of the population density obtained by mapping the positions of the ligand center of mass (COM) in all frames of the trajectories onto a 3D grid. Helices D, F and G are shown in pink, yellow and blue, respectively.*

**
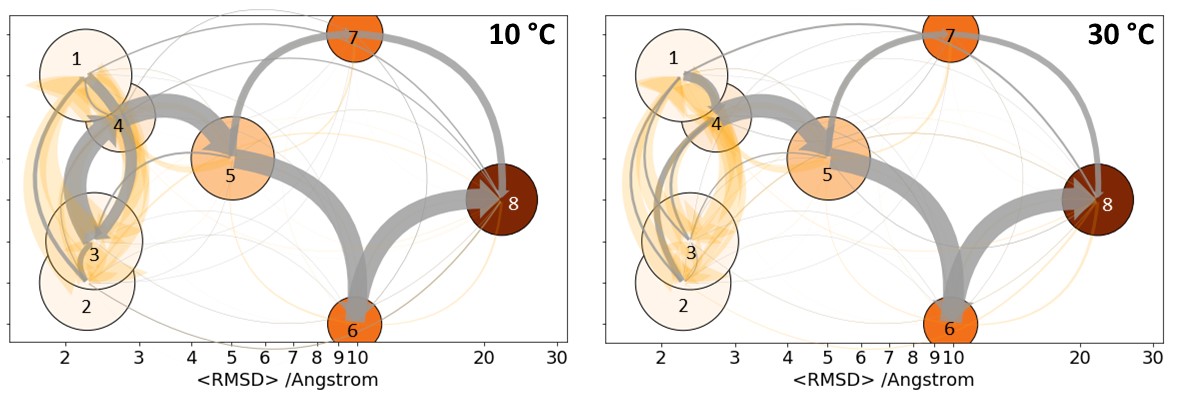
**

***Figure S6.*** *Analysis of egress routes for benzene from T4L:L99A at 10 °C (left) and 30 °C (right) in RAMD trajectories. Clusters were defined by clustering of the last 300 frames in each trajectory in the IFP space; simulations at 10, 20 and 30 °C were included in the clustering procedure. Dissociation pathways are shown in a graph representation; each node represents a cluster that is colored and positioned according to increasing mean RMSD of benzene in the cluster from in the starting complex; the node size denotes the cluster population; transitions between nodes are indicated by arrows for simulations: the net transition flux between nodes is shown by gray arrows with their thickness proportional to the flux magnitude; transition between states are shown by orange arrows with their thickness proportional to the transition number.*

**
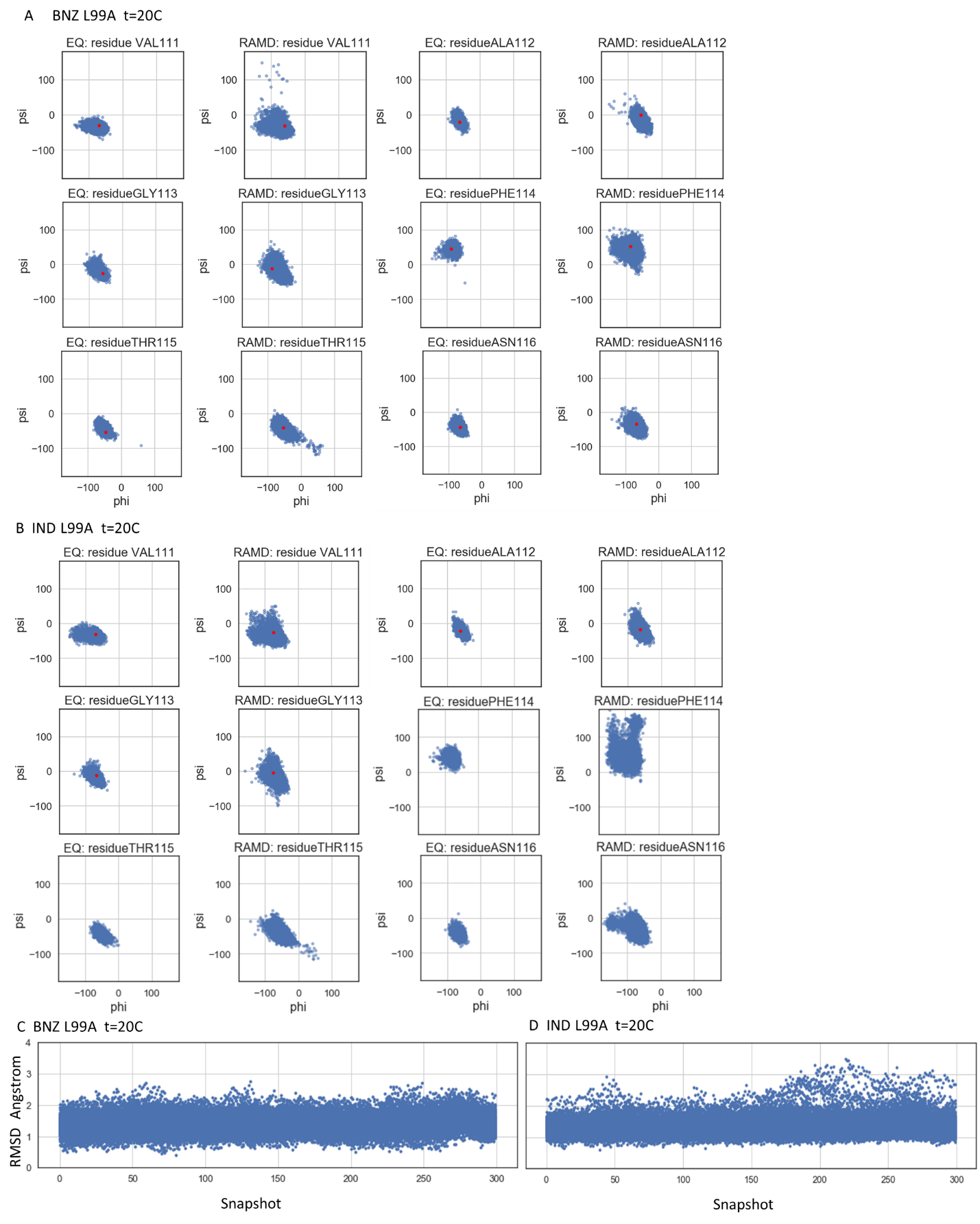
**

***Figure S7.*** *Conformational changes in T4L:L99A upon ligand egress. (A, B) Ramachandran plots for several residues in the region between the F and G helices generated from the equilibration (EQ) and RAMD trajectories (the last 300 snapshots from each trajectory were used in the analysis) of T4L:L99A for the egress of (A) benzene and (B) indole at 20 °C. The red dots show the angles for the corresponding crystal structures. (C, D) RMSD of the non-hydrogen atoms of the residues of helix F (G108 – T115) relative to the starting structure of the T4L:L99A – ligand complex, plotted for the last 300 snapshots of each RAMD trajectory for (C) benzene and (D) indole dissociating from T4L:L99A at 20 °C. Conformational changes in T4L:L99A from a highly to a lowly populated state, characterized by NMR* [17,18]*, were observed to facilitate benzene unbinding through path FGH in several computational studies* [19,20]*, while in other studies, such changes were observed for the binding of bulkier ligands to T4L:L99A, but not for benzene* [21]*. This conformational change mainly involves helix F and one of its features is a change in the F114 psi angle from 50° to -40°. (A, C) show that there are no clear conformational changes of the helix F backbone or of F114 upon benzene dissociation that would characterize a transition to a lowly populated state of the protein with a conformation different from that in the bound structure although the protein RMSD and the dihedral angles for residues V111-T115 show greater variation in RAMD dissociation simulations than in equilibration MD. The variations in protein conformation are notably greater in the case of indole dissociation (B, D), which is in line with the results of Ref.* [21] *showing that protein distortion is caused by ligands larger than benzene.*


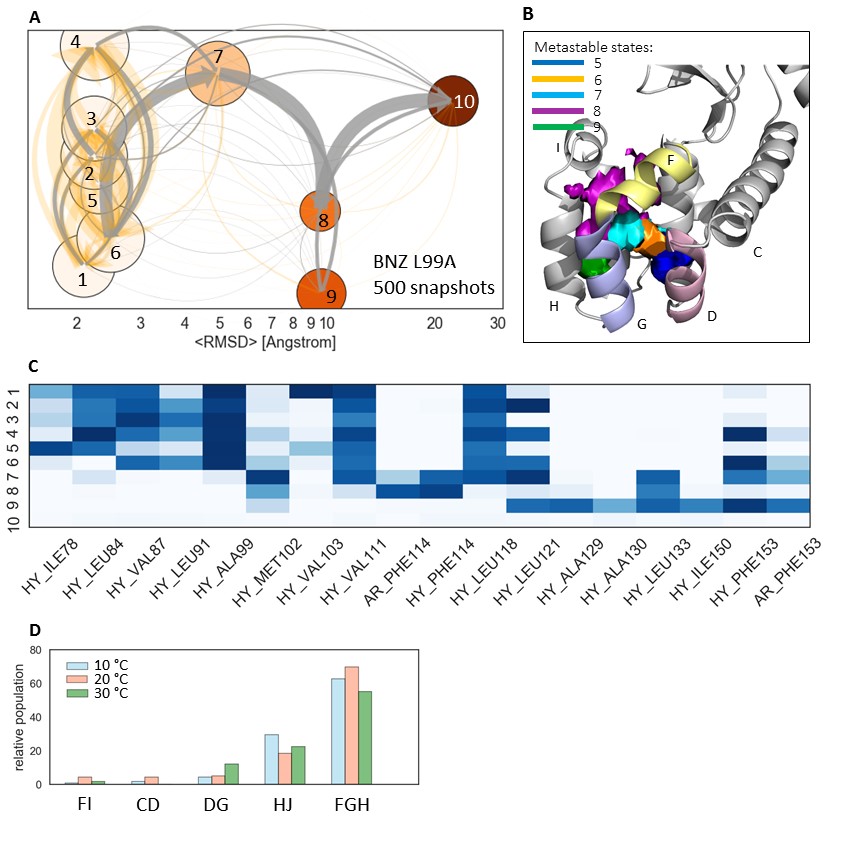


***Figure S8.*** *Analysis of benzene unbinding from T4L:L99A in IFP space using the last 500 snapshots of each RAMD trajectory. Simulations at temperatures of 10, 20 and 30 °C and pH 5.5 were included in the clustering procedure. (A) Dissociation pathways are shown in a graph-representation; each node represents a cluster or metastable state that is colored and placed according to increasing mean RMSD of benzene in the cluster from in the starting complex; the node size denotes the cluster population; transitions between nodes are indicated by arrows for simulations at 20 °C: the net transition flux between nodes is shown by gray arrows with thickness proportional to the flux magnitude; transitions between states are shown by orange arrows with the thickness proportional to the transition number. (B) The clusters shown in (A) are displayed on the T4L:L99A structure; selected clusters are shown by isosurfaces of the ligand COM population density mapped onto a 3D grid; helices D, F and G are shown in pink, yellow and blue, and benzene is shown in ball and stick representation in cyan in its bound position. (C) Cluster composition, shown as contacts between T4L:L99A and benzene and ordered by benzene RMSD. (D) Relative pathway populations observed in simulations at the three different temperatures of 10, 20 and 30 °C.*

**References**

1. Liu L, Marwitz AJV, Matthews BW, Liu S-Y: **Boron Mimetics: 1,2-Dihydro-1,2-azaborines Bind inside a Nonpolar Cavity of T4 Lysozyme**. *Angew Chemie Int Ed* 2009, **48**:6817–6819.

2. Baldwin E, Baase WA, Zhang X, Feher V, Matthews BW: **Generation of ligand binding sites in T4 lysozyme by deficiency-creating substitutions**. *J Mol Biol* 1998, **277**:467–485.

3. Morton A, Matthews BW: **Specificity of ligand binding in a buried nonpolar cavity of T4 lysozyme: Linkage of dynamics and structural plasticity**. *Biochemistry* 1995, **34**:8576–8588.

4. Dolinsky TJ, Nielsen JE, McCammon JA, Baker NA: **PDB2PQR: An automated pipeline for the setup of Poisson-Boltzmann electrostatics calculations**. *Nucleic Acids Res* 2004, **32**:W665-7.

5. Dolinsky TJ, Czodrowski P, Li H, Nielsen JE, Jensen JH, Klebe G, Baker NA: **PDB2PQR: Expanding and upgrading automated preparation of biomolecular structures for molecular simulations**. *Nucleic Acids Res* 2007, **35**:W522–W525.

6. Feher VA, Baldwin EP, Dahlquist FW: **Access of ligands to cavities within the core of a protein is rapid**. *Nat Struct Biol* 1996, **3**:516–521.

7. Kokh DB, Amaral M, Bomke J, Grädler U, Musil D, Buchstaller H-P, Dreyer MK, Frech M, Lowinski M, Vallee F, et al.: **Estimation of drug-target residence times by τ-random acceleration molecular dynamics simulations**. *J Chem Theory Comput* 2018, **14**:3859–3869.

8. Maier JA, Martinez C, Kasavajhala K, Wickstrom L, Hauser K, Simmerling C, Hauser KE: **ff14SB: Improving the accuracy of protein side chain and backbone parameters from ff99SB ff14SB: Improving the accuracy of protein side chain and backbone parameters from ff99SB**. *J Chem Theory Comput* 2015, **11**:3696–3713.

9. Wang J, Wolf RM, Caldwell JW, Kollman PA, Case DA: **Development and Testing of a General Amber Force Field**. *J Comput Chem* 2004, **25**:1157–1174.

10. Bayly CI, Cieplak P, Cornell WD, Kollman’ PA: **A Well-Behaved Electrostatic Potential Based Method Using Charge Restraints for Deriving Atomic Charges: The RESP Model**. *J Phys Chem* 1993, **97**:10269–10280.

11. Cornell WD, Cieplak P, Bayly CI, Kollman PA: **Application of RESP charges to calculate conformational energies, hydrogen bond energies, and free energies of solvation**. *J Am Chem Soc* 1993, **115**:9620–9631.

12. Gordon MSMS, Schmidt MWMW: *Chapter 41 – Advances in electronic structure theory: GAMESS a decade later*. Elsevier; 2005.

13. D.A. Case, V. Babin, J.T. Berryman, R.M. Betz, Q. Cai, D.S. Cerutti, T.E. Cheatham, III, T.A. Darden, R.E. Duke, H. Gohlke, A.W. Goetz, S. Gusarov, N. Homeyer, P. Janowski, J. Kaus, I. Kolossváry, A. Kovalenko, T.S. Lee, S. LeGrand, T. Luchko, R. Luo, B. XW and PAK: **“AMBER 14”, University of California, San Francisco**. 2014,

14. Phillips JC, Braun R, Wang W, Gumbart J, Tajkhorshid E, Villa E, Chipot C, Skeel RD, Kalé L, Schulten K: **Scalable molecular dynamics with NAMD**. *J Comput Chem* 2005, **26**:1781–1802.

15. Lüdemann SK, Lounnas V, Wade RC: **How do substrates enter and products exit the buried active site of cytochrome P450cam? 1. Random expulsion molecular dynamics investigation of ligand access channels and mechanisms**. *J Mol Biol* 2000, **303**:797–811.

16. Kokh DB, Doser B, Richter S, Ormersbach F, Cheng X, Wade RC: **A Workflow for Exploring Ligand Dissociation from a Macromolecule: Efficient Random Acceleration Molecular Dynamics Simulation and Interaction Fingerprints Analysis of Ligand Trajectories**. *J Chem Phys* 2020, **153**:125102.

17. Bouvignies G, Vallurupalli P, Hansen DF, Correia BE, Lange O, Bah A, Vernon RM, Dahlquist FW, Baker D, Kay LE: **Solution structure of a minor and transiently formed state of a T4 lysozyme mutant**. *Nature* 2011, **477**:111–117.

18. Mulder FAA, Mittermaier A, Hon B, Dahlquist FW, Kay LE: **Studying excited states of proteins by NMR spectroscopy**. *Nat Struct Mol Biol* 2001, **8**:932–935.

19. Feher VA, Schiffer JM, Mermelstein DJ, Mih N, Pierce LCT, McCammon JA, Amaro RE: **Mechanisms for Benzene Dissociation through the Excited State of T4 Lysozyme L99A Mutant.** *Biophys J* 2019, **116**:205–214.

20. Wang Y, Papaleo E, Lindorff-Larsen K: **Mapping transiently formed and sparsely populated conformations on a complex energy landscape**. *Elife* 2016, **5**:e17505.

21. Niitsu A, Re S, Oshima H, Kamiya M, Sugita Y: **De Novo Prediction of Binders and Nonbinders for T4 Lysozyme by gREST Simulations**. *J Chem Inf Model* 2019, **59**:3879–3888.
